# Aberrant mitochondrial dynamics contributes to diaphragmatic weakness induced by mechanical ventilation

**DOI:** 10.1093/pnasnexus/pgad336

**Published:** 2023-11-07

**Authors:** Haikel Dridi, Marc Yehya, Robert Barsotti, Yang Liu, Steven Reiken, Lan Azria, Qi Yuan, Laith Bahlouli, Rajesh Kumar Soni, Andrew R Marks, Alain Lacampagne, Stefan Matecki

**Affiliations:** Department of Physiology and Cellular Biophysics, Clyde and Helen Wu Center for Molecular Cardiology, NewYork, NY 10032, USA; Department of Medicine, Columbia University Vagelos College of Physicians and Surgeons, NewYork, NY 10032, USA; PhyMedExp, INSERM, CNRS, University of Montpellier, Montpellier 34000, France; Department of Biomedical Sciences, Philadelphia College of Osteopathic Medicine, Philadelphia, PA 19131, USA; Department of Physiology and Cellular Biophysics, Clyde and Helen Wu Center for Molecular Cardiology, NewYork, NY 10032, USA; Department of Medicine, Columbia University Vagelos College of Physicians and Surgeons, NewYork, NY 10032, USA; Department of Physiology and Cellular Biophysics, Clyde and Helen Wu Center for Molecular Cardiology, NewYork, NY 10032, USA; Department of Medicine, Columbia University Vagelos College of Physicians and Surgeons, NewYork, NY 10032, USA; PhyMedExp, INSERM, CNRS, University of Montpellier, Montpellier 34000, France; Department of Physiology and Cellular Biophysics, Clyde and Helen Wu Center for Molecular Cardiology, NewYork, NY 10032, USA; Department of Medicine, Columbia University Vagelos College of Physicians and Surgeons, NewYork, NY 10032, USA; Department of Physiology and Cellular Biophysics, Clyde and Helen Wu Center for Molecular Cardiology, NewYork, NY 10032, USA; Department of Medicine, Columbia University Vagelos College of Physicians and Surgeons, NewYork, NY 10032, USA; Proteomics and Macromolecular Crystallography Shared Resource, Herbert Irving Comprehensive Cancer Center, NewYork, NY 10032, USA; Department of Physiology and Cellular Biophysics, Clyde and Helen Wu Center for Molecular Cardiology, NewYork, NY 10032, USA; Department of Medicine, Columbia University Vagelos College of Physicians and Surgeons, NewYork, NY 10032, USA; PhyMedExp, INSERM, CNRS, University of Montpellier, Montpellier 34000, France; PhyMedExp, INSERM, CNRS, University of Montpellier, Montpellier 34000, France

**Keywords:** mitochondrial fission, dynamin-related protein 1, FIS1 inhibitor, calcium homeostasis

## Abstract

In critical care patients, the “”temporary inactivity of the diaphragm caused by mechanical ventilation (MV) triggers a series of events leading to diaphragmatic dysfunction and atrophy, commonly known as ventilator-induced diaphragm dysfunction (VIDD). While mitochondrial dysfunction related to oxidative stress is recognized as a crucial factor in VIDD, the exact molecular mechanism remains poorly understood. In this study, we observe that 6 h of MV triggers aberrant mitochondrial dynamics, resulting in a reduction in mitochondrial size and interaction, associated with increased expression of dynamin-related protein 1 (DRP1). This effect can be prevented by P110, a molecule that inhibits the recruitment of DRP1 to the mitochondrial membrane. Furthermore, isolated mitochondria from the diaphragms of ventilated patients exhibited increased production of reactive oxygen species (ROS). These mitochondrial changes were associated with the rapid oxidation of type 1 ryanodine receptor (RyR1) and a decrease in the stabilizing subunit calstabin 1. Subsequently, we observed that the sarcoplasmic reticulum (SR) in the ventilated diaphragms showed increased calcium leakage and reduced contractile function. Importantly, the mitochondrial fission inhibitor P110 effectively prevented all of these alterations. Taken together, the results of our study illustrate that MV leads, in the diaphragm, to both mitochondrial fragmentation and dysfunction, linked to the up-/down-regulation of 320 proteins, as assessed through global comprehensive quantitative proteomics analysis, primarily associated with mitochondrial function. These outcomes underscore the significance of developing compounds aimed at modulating the balance between mitochondrial fission and fusion as potential interventions to mitigate VIDD in human patients.

**Significance Statement**

Mechanical ventilation (MV) is a double-edged sword. Although life-saving for patients in intensive care unit, MV induces diaphragm weakness. This side effect appears to decrease their ability to be successfully weaned from the ventilator with a major impact on clinical practice, patient's morbidity, and the healthcare resource utilization. We have identified the presence of a mitochondrial fragmentation in the diaphragm, induced by MV. We have also observed that this fragmentation, called fission, leads to dysfunctional mitochondrial with production of pathological oxygen species. This results in an impaired ability of diaphragmatic muscle fibers to modulate intracellular calcium fluxes, leading to their weakness. We finally demonstrate that prevention of mitochondrial fission may provide a novel therapeutic approach to prevent MV-induced diaphragm weakness.

## Introduction

A substantial portion of critically ill patients admitted to the intensive care unit for acute respiratory failure necessitating mechanical ventilation (MV) experience diaphragm dysfunction.

The use of MV leads to sudden diaphragm contractile inactivity and weakness, which can appear as early as the first few days [1]. The molecular events involved in ventilator-induced diaphragm dysfunction (VIDD) include activation of apoptosis, autophagy, Ca^2+^ homeostasis disturbance, and calpain activation [2–6]. Most of these subcellular events are triggered by increased oxidative stress due to diaphragm inactivity [2–4], highlighting the interest of using antioxidant molecules to prevent VIDD [5].

Indeed, it has been reported that mitochondria are the main source of reactive oxygen species (mROS) generation during VIDD but the mechanism triggering mROS production is poorly known [6, 7].

Mitochondria are vital cellular organelles whose structure and role are intricately intertwined. Well-coordinated fusion and fission processes sculpt mitochondria to meet metabolic requirements and facilitate the removal of impaired organelles [8]. The acute diaphragm inactivity induced by MV may provoke metabolic substrate overload with exacerbated oxidative stress [2] associated with mitochondria fragmentation [9, 10]. However, the role of mitochondria dynamics in VIDD, as a pathological mechanism as well as a potential therapeutic target, remains to be elucidated.

Interestingly, mROS production, which is a main cellular mechanism implicated in the development of VIDD [11], has been also associated with an exacerbated mitochondrial fission process [9].

The mitochondrial dynamic apparatus comprises a group of shaping proteins often referred to as the dynamin-like guanosine triphosphatases family (GTPases), which facilitate the fusion and fission of mitochondrial membranes [12]. Mitofusins 1 and 2 (MFN1 and MFN2) oversee the fusion of the outer mitochondrial membrane [13], whereas OPA1 is required for inner mitochondrial membrane fusion [14].

Fission, on the other hand, is executed by dynamin-related protein 1 (DRP1) [12], a cytosolic protein that is recruited to the mitochondrial surface, by binding receptor FIS1 (mitochondrial fission 1 protein: an outer mitochondrial membrane receptor), in response to various physiological cues.

In a state of mitochondrial fission/fusion equilibrium, DRP1 is evenly distributed in the cytoplasm, with some DRP1 molecules localized to specific punctate foci on mitochondria.

When there is a transition to extensive mitochondrial fission, DRP1 is markedly recruited to the outer mitochondrial membrane, forming multi-unit rings responsible for constricting and dividing the mitochondrial tubule into two smaller mitochondria [15].

The recruitment of cytosolic DRP1 to this organelle is facilitated through a variety of DRP1 binding receptor proteins located on the outer mitochondrial membrane, including FIS1 [16, 17].

We hypothesized that inhibiting the interaction between DRP1 and its receptor FIS1 may limit mitochondrial fission, mROS production, and the subsequent diaphragmatic dysfunction induced by MV. To test this hypothesis, we used a cell permeable peptide called P110 that inhibits mitochondrial fragmentation by blocking DRP1 binding to its mitochondrial receptor FIS1 [18].

Mitochondria are situated in close proximity to the redox-sensitive ryanodine receptor type 1 in the sarcoplasmic reticulum (SR), which is responsible for calcium (Ca2+) release.

Its MV-induced oxidation results in abnormal Ca^2+^ leak from the SR and contractile dysfunction, and has been proposed as an early event involved in VIDD [19]. It has been shown that with longer periods of MV, this RyR1 remodeling leads to impaired Ca^2+^ handling, secondary activation of the calpain pathway, and finally atrophy [19]. We recently observed that in mice, limiting mROS production with SS-31 (a mitochondrial targeted antioxidant peptide) during MV reduces the depletion of calstabin1 from RyR1, prevents the SR Ca^2+^ leak, and preserves diaphragm strength [20].

Therefore, to evaluate whether mitochondrial dynamics can be considered as an upstream cellular pathway to Ca^2+^ homeostasis impairment, thereby a more effective therapeutic target, we first performed, for the first time in mechanically ventilated mice, diaphragm proteomic profiling to verify if MV may alter mitochondrial pathways related to mitochondrial fission and adenosine triphosphate (ATP) synthesis. Then we evaluated whether preventing mitochondrial fragmentation with P110 may attenuate mROS production and sufficiently limit RyR1 oxidation as well as the subsequent Ca^2+^ leak that underlies the development of VIDD.

## Results

### Six hours of MV induces deep changes to the diaphragmatic proteomics including those involved in mitochondrial function

**Fig. 1.**
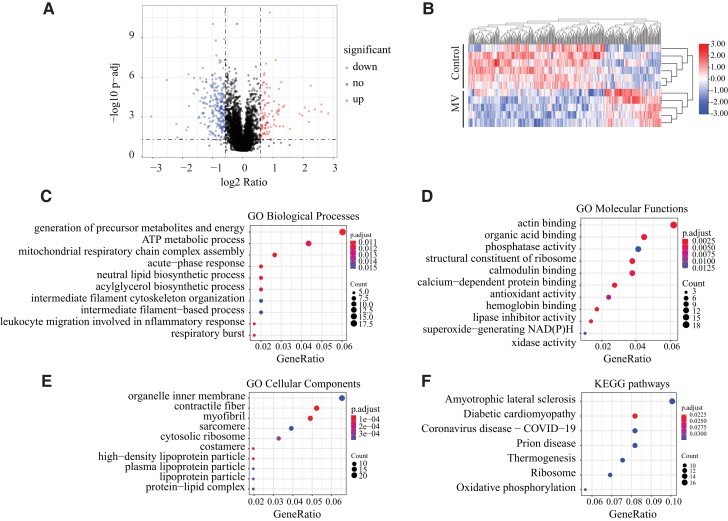
Quantitative proteomics analysis in controls and mechanically ventilated mice. **A)** Quantitative proteomics was performed on samples from diaphragms of controls (*n* = 6) and MV (*n* = 5). The Volcano plot shows differentially expressed proteins (*P*-adj < 0.05, fold-change ≥ 1.5) in controls and MV. Red indicates up-regulation, while blue represents down-regulation of protein expression. Black indicates unchanged expression levels. **B)** The heat map of significantly dysregulated proteins (down-regulated: 225, up-regulated: 94). Red represents up-regulated proteins, while the blue shows the down-regulated proteins. **C)** Dot plots show top 10 GO BP, **D)** MF, **E)** CC, and **F)** KEGG pathways that were enriched from differentially expressed proteins. See Table S1 for proteins list.

We followed an unbiased discovery approach to evaluate whether mitochondria are a major contributor to VIDD using free labeled proteomic analysis of the whole cell lysates isolated from diaphragms of controls and 6-h mechanically ventilated mice. We obtained 4,129 proteins with at least two unique peptides and 1% false discovery rate (FDR). A volcano plot of all of these proteins is displayed in Fig. 1A. Based on the criteria of adjusted *P*-value < 0.05, fold-change ≥ 1.5, and unique peptide ≥ 1, 319 differentially expressed unique proteins (control vs. MV) were identified and selected for further analysis (Table S1). Among these, 225 proteins were down-regulated and 94 were up-regulated. The heatmap of 319 differentially expressed proteins is shown in Fig. 1B. The control and MV groups were separately clustered, and the 6-5 replicated of each group (respectively) showed good reproducibility. We then performed gene ontology (GO) enrichment analysis of these changed proteins. The top 10 significant GO terms of biological process (BP), molecular function (MF), and cellular component (CC) are shown in Fig. 1C–E.

The BP GO analysis indicates that the proteins with differential expression were primarily associated with the following terms: generation of precursor metabolites and energy, ATP metabolic process, and assembly of the mitochondrial respiratory chain complex (Fig. 1C). The MFs of these regulated proteins are mainly related to actin binding, organic acid binding, phosphatase activity, and Ca^2+^ dependent protein binding (Fig. 1D). The significantly altered proteins expressions were located mainly at the organelle inner membrane and the contractile fibers (Fig. 1E). We also analyzed Kyoto Encyclopedia of Genes and Genomes (KEGG) pathways and found significant enrichment of amyotrophic lateral sclerosis and oxidative phosphorylation (Fig. 1F).

**Fig. 2.**
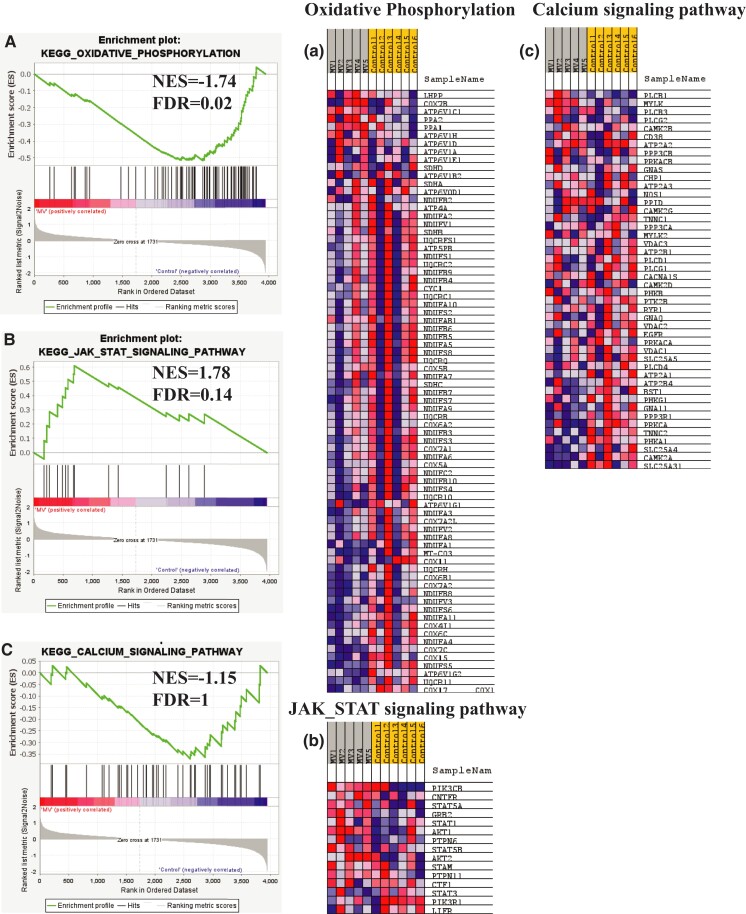
GSEA of the diaphragmatic proteomics. The enrichment plots of representative KEGG pathway gene sets demonstrate oxidative phosphorylation (**panel A**; NES = −1.74; FDR = 0.02), JAK-STAT (**panel B;** NES = 1.78; FDR = 0.14), and calcium signaling pathway (**panel C**; NES = −1.15; FDR = 1) with a, b, and c as the heatmaps of proteins involved in each of these pathways. The heatmap on the right side of each panel visualizes the genes contributing to the enriched pathways. For the detailed list, see Table S2. Signal-to-noise ratio was used to rank the genes per their correlation with either MV phenotype (red) or control phenotype (blue). The *y*-axis represents enrichment score (ES) and on the *x*-axis are genes (vertical black lines) represented in gene sets. The GSEA analysis calculates an ES (the maximum deviation from zero) reflecting the degree of over-representation of a gene set at the top or the bottom of the ranked gene list. A positive ES indicates gene set enrichment at the top of the ranked list; a negative ES indicates gene set enrichment at the bottom of the ranked list. NES: normalized enrichment score; FDR: FDR adjusted *P*-value.

Next, we performed gene set enrichment analyses (GSEAs) with the canonical pathway and GO gene sets (Fig. 2A–C and a–c). Interestingly, there was enrichment of GO terms related to Ca^2+^ signaling and the Janus kinase/signal transducers and activators of transcription (JAK-STAT) signaling pathway, previously reported in VIDD [21]. Interestingly, oxidative phosphorylation GO term was down-regulated (Fig. 2A/a). In further detail, the depressed oxidative phosphorylation pathway in MV was mainly due to several reductions in the expression levels of the different subunits of cytochrome c oxidase and ubiquinone oxidoreductase, the main translocator of protons across the inner mitochondrial membrane. With regard to the dysregulated Ca^2+^ homeostasis pathways, RyR1 and ATPase sarcoplasmic/endoplasmic reticulum Ca^2+^ transporting were down-regulated while STAT3, STAT5B, and STAT1 (major components of the JAK-STAT pathway) were up-regulated following MV in comparison to controls, which confirms their implication in VIDD (Table S2).

### DRP1 inhibitor P110 prevented mitochondrial morphology disruption

**Fig. 3.**
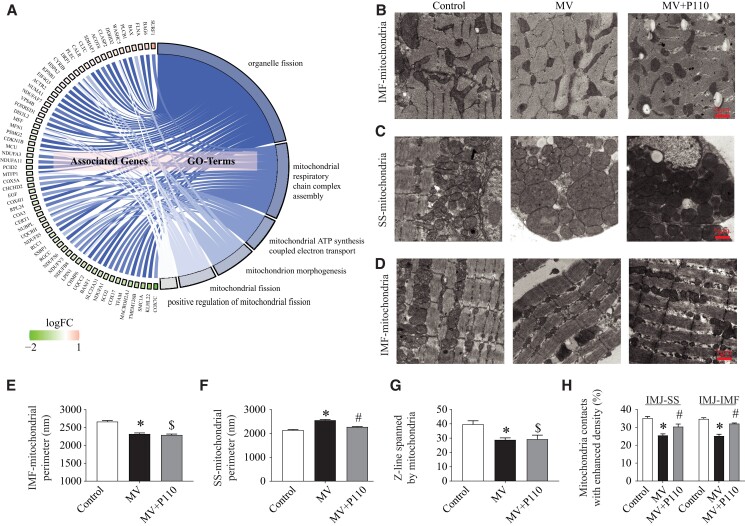
P110 inhibitor reduces mitochondrial fission induced by MV. **A)** Cohort plot representation of differentially expressed mitochondrial proteins (MV vs. controls, FDR 0.05) from top five enriched mitochondrial pathways and generated by GOplot. The color map represents fold-change of proteins (log_2_ scale). **B)** Representative electron micrographs of IMF mitochondria from control, MV, and MV + P110 diaphragms in transverse orientation. **C)** Representative electron micrographs of SS mitochondria from control, MV, and MV + P110 diaphragms in longitudinal orientation. **D)** Representative electron micrographs of IMF mitochondria from control, MV, and MV + P110 diaphragms in longitudinal orientation. **E)** IMF mitochondria perimeter in the three experimental groups. **F)** SS mitochondria perimeter in the three experimental groups. **G)** Proportion of IMF-mitochondria interacting across Z-lines in control, MV, and MV + P110 diaphragms. **H)** Proportion of mitochondria IMJ in IMF and SS mitochondria in control, MV, and MV + P110 diaphragms. Scale bars = 1 µm. *N* = 6 per group. Data are mean ± SEM. One-way ANOVA shows *P < 0.05 control vs. MV, ^#^*P* < 0.05 MV vs. MV + P110, ^$^*P* < 0.05 control vs. MV + P110.

As shown in Fig. 3A, the differentially expressed mitochondrial proteins (MV vs. controls, FDR 0.05) from the top five enriched mitochondrial pathways were related to mitochondrial respiratory chain complex assembly, ATP synthesis, and mitochondrial fission. Moreover, DRP1 was in the top up-regulated proteins list whereas MFN1 was down-regulated. Based on these proteomic findings, we hypothesize that this dysregulation of the mitochondrial shaping proteins would result in significant mitochondrial structural and functional damage. Therefore, we looked at the mitochondrial morphology in controls as well as ventilated diaphragms with or without an inhibitor of mitochondrial fragmentation, P110, using electron microscopy (EM). Electron micrographs of thin sections with myofilaments oriented either longitudinally or transversally were collected from all three groups. Mitochondrial interaction was visualized as electron-dense intermitochondrial junction (IMJ) between mitochondrial membranes. Representative examples of intermyofibrillar (IMF) mitochondria in transverse and longitudinal sections from control, MV, and MV treated with P110 are shown, respectively, in Fig. 3B–D. IMF mitochondria in the control diaphragm show an elongated and wavy serpentine structure whereas IMF mitochondria in the MV group are shorter and show less branching and morphogical complexity (Fig. 3B, E, and H). P110 treatment prevented the MV-induced reduction of IMJ between IMF mitochondria induced by MV without any effect on their perimeter (Fig. 3E).

Mitochondrial interactions are commonly observed in pairs, arranged longitudinally and flanking the Z-lines, with less frequent occurrences as continuous organelles spanning across the Z-line.

Thus, as shown in Fig. 3D and G, we observed that MV was associated with a decreased percentage of mitochondria crossing Z-lines in the IMF population without any significant effect of P110 treatment on this mitochondrial interaction parameter.

Subsarcolemmal (SS) mitochondria were only observed in a longitudinal orientation (Fig. 3C). SS mitochondria in the MV group diaphragm show a larger size and more roundness with less connection between each other (Fig. 3C, F, and H). The increase of mitochondrial size in SS mitochondria with weak branching (Fig. 3H) under these conditions could be interpreted as mitochondria swelling and evaluated herein by measuring the mitochondrial perimeter as previously reported [22]. After 6 h of MV, we observed that P110 treatment prevented the increased SS mitochondrial perimeter (Fig. 3C and F) and enhanced the proportion of IMJ between SS mitochondria (Fig. 3H).

### P110 treatment decreased MV-induced DRP1 recruitment to mitochondria

P110 is a cell permeable peptide that inhibits mitochondrial fragmentation by blocking DRP1 binding to its mitochondrial receptor FIS1. We therefore evaluated the effect of P110 on the aforementioned DRP1-FIS1 interaction in the MV diaphragms. We observed that P110 successfully abolished DRP1 recruitment to the mitochondria compared to the MV untreated group in both the SS and IMF mitochondrial populations (Fig. S1A–D).

To investigate the potential role of P110 in the regulation of other major mitochondrial shaping proteins involved in mitochondrial dynamics, we evaluated the total levels of the MFN1 and MFN2 ratio in MV diaphragms. We observed that MV significantly decreased MFN1 expression levels but not MFN2. P110 treatment had no significant effect on either protein's expression levels (Fig. S1E–G). Moreover, we evaluated the diaphragm expression of short and long forms of OPA1 in our three experimental groups.

Indeed, the dynamin-like GTPase OPA1 serves as a mediator of mitochondrial fusion and plays a crucial role in shaping mitochondrial cristae morphology and enhancing resistance to apoptosis [23].

Within the inner mitochondrial membrane, two peptidases, namely OMA1 and the I-AAA protease YME1L, convert long OPA1 forms (L-OPA1) into shorter forms (S-OPA1) [24].

The fusion of mitochondria relies on L-OPA1, whereas S-OPA1 is linked to mitochondrial fission [24]. We observed a total expression decrease of OPA1 induced by 6 h of MV without any preventive effect of P110 treatment. However, our results did not show any significant proteolytic conversion between L-OPA1 and S-OPA1 across all the groups (Fig. S1E–J).

### Effect of DRP1 inhibition on mitochondrial function

**Fig. 4.**
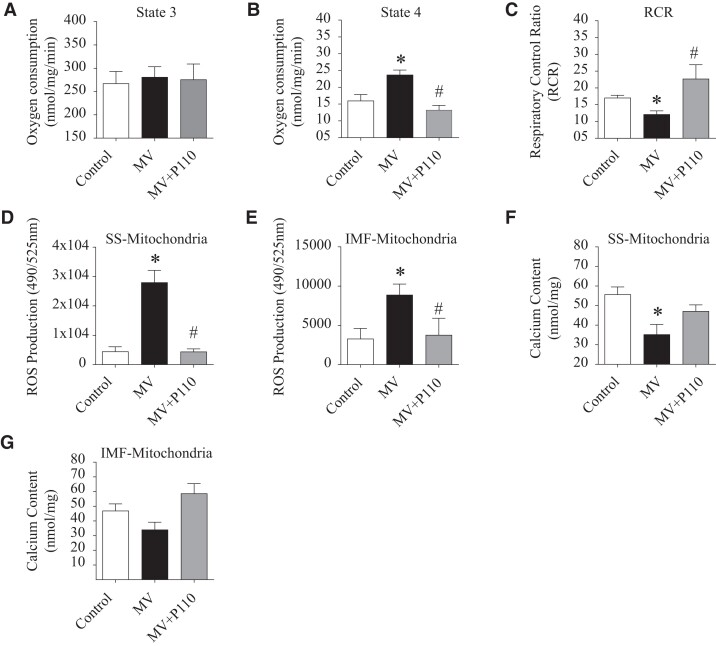
Effect of P110 inhibitor on mitochondrial function. **A** and **B)** Mitochondrial oxygen consumption (nmol/mg/min) at states 3 and 4 in diaphragmatic tissues of controls (*n* = 7), MV (*n* = 8), and MV + P110 (*n* = 7). **C)** Mitochondrial RCR in the same experimental groups. **D** and **E)** SS mitochondrial and intramyofibrillar (IMF) mROS production in the same experimental groups. **F** and **G)** SS and IMF mitochondrial calcium content (nmol/mg) in controls, MV, and MV + P110 groups. Data are mean ± SEM. One-way ANOVA shows *P < 0.05 control vs. MV, ^#^*P* < 0.05 MV vs. MV + P110.

Mitochondrial fission is associated with an increased number of damaged mitochondria and a subsequent increase in mROS production, and a decrease in oxygen consumption [10]. To understand the link between these events, we isolated diaphragm mitochondria from our three experimental groups and evaluated mitochondrial coupling efficacy and the extent of reactive oxygen species (ROS) production. We observed a significant increase of mitochondrial oxygen consumption rates in the MV group compared to controls at state 4 whereas no significant changes were observed at state 3 (Fig. 4A and B). Moreover, the mitochondrial respiratory control ratio (RCR) was significantly decreased in the ventilated diaphragms compared to unventilated (Fig. 4C). Of note, we were not able to analyze these two functional parameters separately for each mitochondrial subpopulation (IMF and SS) due to technical limits (low SS mitochondrial yield). However, we measured mitochondrial ROS production which was significantly increased in both IMF and SS mitochondria subpopulations after 6 h of MV (Fig. 4D and E). Interestingly, DRP1 inhibitor, P110, restored normal mitochondrial respiration and blunted the excessive ROS production induced by MV. Finally, we evaluated mitochondrial Ca^2+^ content, changes in which could be linked to the mitochondrial morphological alterations. We observed that the mitochondrial Ca^2+^ level was decreased mainly in the SS subpopulation (Fig. 4F and G). One explanation would be the considerable dissipation of the mitochondrial membrane potential and the release of the mitochondrial content (see discussion).

### Downstream of mitochondrial dysfunction: P110 prevents RyR1 remodeling and Ca^2+^ leak

**Fig. 5.**
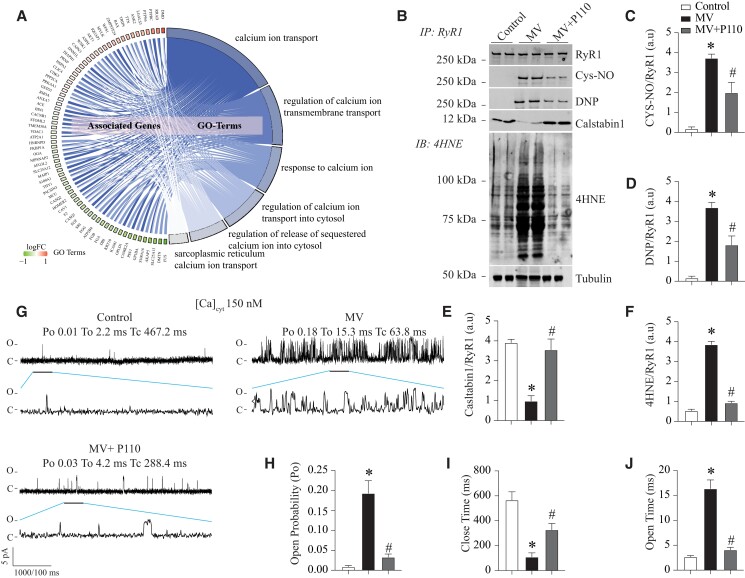
Effect of P110 on RyR1 remodeling and function in diaphragms submitted to MV. **A)** Cohort plot representation of differentially expressed Ca^2+^ related proteins (MV vs. controls, FDR 0.05) from top six enriched Ca^2+^ pathways and generated by GO plot. The color map represents fold-change of proteins (log_2_ scale). **B)** Representative immunoblots of immunoprecipitated RyR1, RyR1 posttranslational modifications (Cys-NO, oxidation (DNP) and bound calstabin1), and total protein oxidation (4HNE) from mouse diaphragm of the three experimental groups: control, MV, and MV + P110. Each blot corresponds to adjacent wells of the same gel. Bar graphs **(C–F)** show quantification of immunoblots, relative to total RyR1 immunoprecipitated and total protein oxidation normalized to Tubulin. Results are expressed as mean ± SEM, *n* = 6 in each group (*P < 0.05 MV vs. control, ^#^MV vs. MV + P110). **G)** Single-channel traces of RyR1 incorporated in planar lipid bilayers with 150 nM Ca^2+^ in the cis chamber, corresponding to representative experiments performed with diaphragm samples from control, MV, and MV + P110 mice. **H)** RyR1 open probability in diaphragm samples from control, MV, and MV + P110 mice. **I)** RyR1 close time (ms) in diaphragm samples from control, MV, and MV + P110 mice. *N* = 5 per group. **J**) RyR1 open time (ms) in diaphragm samples from control, MV, and MV + P110 mice. Data are mean ± SEM. One-way ANOVA shows *P < 0.05 control vs. MV, ^#^*P* < 0.05 MV vs. MV + P110.

Recent suggestions point toward the remodeling of the SR calcium release channel, known as ryanodine receptors (RyR1), in the diaphragm induced by mitochondrial ROS production [20] and is a proximal mechanism of VIDD in humans, mice, and piglets [19, 25]. Following the oxidation of RyR1, the association between calstabin1 and the rest of the RyR1 complex is lost [26], leading to leaky RyR1 channels which cause impaired Ca^2+^ homeostasis and contractile dysfunction. Herein, we suggest that MV, which alters mitochondrial dynamics, increases mROS production leading thereby to RyR1 channels' oxidation and exacerbation of the pathological SR Ca^2+^ leak. These results are in accordance with differentially expressed proteins related to Ca^2+^ ion transport, release, and sequestration observed in the ventilated diaphragms (Fig. 5A). Furthermore, we analyzed the composition of the RyR1 macromolecular complex and posttranslational modifications known to be associated with the channels' Ca^2+^ leak. RyR1 channels from mechanically ventilated diaphragm were oxidized, nitrosylated, and depleted of calstabin1 compared to control, which is the biochemical signature of leaky RyR1 channels. Total diaphragmatic protein oxidation levels were increased as assessed by 4-hydroxy-2-nonenal (4HNE) oxidative marker (Fig. 5B–F). When single-channel recordings of diaphragmatic RyR1, reconstituted into planar lipid bilayers, were conducted, it was observed that the open probability (P0) increased in the presence of low, non-activating [Ca2+]_cis_ conditions. Under these conditions, normal RyR1 channels remain tightly closed.

This elevated that P_0_ is consistent with pathological SR Ca^2+^ leak. Interestingly, P110 treatment reduced RyR1 oxidation, enhanced calstabin1 binding to the channel, reduced the SR Ca^2+^ leak, and reduced total proteins oxidation (Fig. 5G–J).

### P110 prevented diaphragmatic weakness induced by MV

**Fig. 6.**
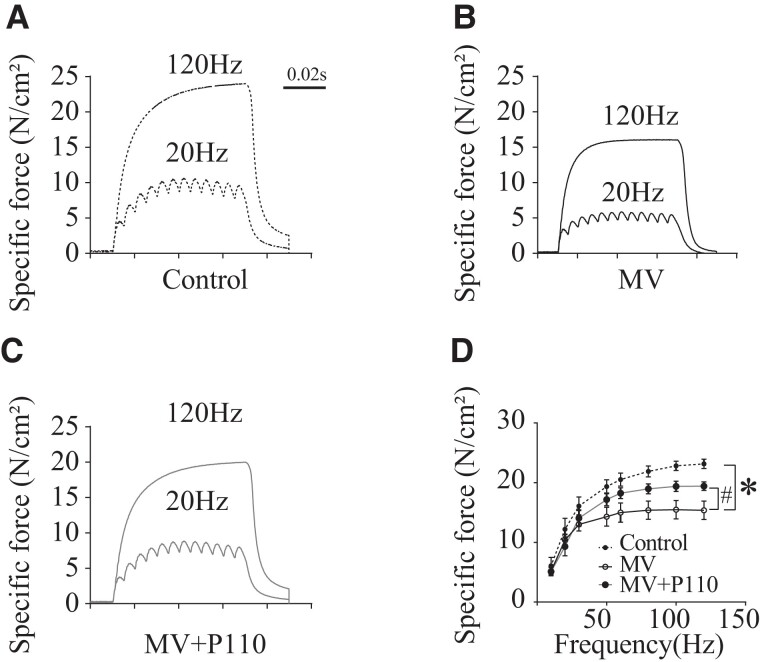
P110 reduces diaphragmatic dysfunction induced by 6 h of MV. **A–C)** Representative records of diaphragmatic specific force production measured *ex vivo* at 20 and 120 Hz in muscle bundles under isometric conditions in wild type (WT), MV, and MV + P110 treated mice. **D)** Average force–frequency relationship recorded in WT, MV, and MV + P110 treated mice. Results are expressed as mean ± SEM (*n* = 6 in each group) (*P < 0.05 vs. control, ^#^MV vs. MV + P110). Data are mean ± SEM. Two-way ANOVA shows *P < 0.05 control vs. MV, ^#^*P* < 0.05 MV vs. MV + P110, ^$^*P* < 0.05 control vs. MV + P110.

To evaluate the overall effect of P110 treatment on diaphragmatic contractile function following MV, we measured the force production of diaphragm muscle strips isolated from our control, MV, and MV + P110 groups. As shown in Fig. 6A–D, 6 h of MV was enough to significantly decrease the diaphragm force generation at different stimulation frequencies tested. We also observed that P110 treatment significantly reduced the diaphragm weakness following 6 h of MV.

## Discussion

The dynamic interplay of mitochondria, wherein they regulate their integrity, distribution, and function by engaging in coordinated cycles of fission and fusion, constitutes a key factor in maintaining cellular energy balance. Disruptions in mitochondrial dynamics have been linked to various neurodegenerative disorders [27], as well as cardiac [14] and skeletal muscle dysfunction [27].

Recently, it has been suggested that an exaggerated mitochondrial fission process may be an initial event in the development of VIDD, although the precise mechanism remains unclear [9]. In the present study, we found that inhibiting DRP1-FIS1 interaction prevented diaphragmatic weakness induced by 6 h of MV in mice. We used P110 heptapeptide conjugated to TAT47–57 to selectively inhibit the interaction between DRP1 and its mitochondrial receptor FIS1, and evaluate its preventive effects on VIDD [18].

We observed that, in ventilated mice diaphragms, P110 treatment enhanced interactions/connections within the mitochondrial network and reduced mROS production following 6 h of MV. Although the mechanisms by which DRP1 binding to the mitochondria causes ROS production are not completely understood, it is likely related to disruption of oxidative phosphorylation coupling. Indeed, fragmented mitochondria exhibit a disorganized electron transport chain with increased electron leakage and loss of mitochondrial membrane potential [10]. The increased oxygen consumption in state 4, associated with a reduced RCR shown in Fig. 4, supports this hypothesis.

We separately assessed IMF and SS mitochondrial morphological remodeling in the diaphragm induced by MV using quantitative EM. In line with a recent study [9], we observed that both SS and IMF mitochondria from mechanically ventilated mice diaphragms undergo aberrant dynamics. They display a shift of their perimeter and a reduction of their interaction and their capacity to form a “healthy” mitochondrial network. Interestingly, as previously shown [9], we observed that MV induced a reduction of the IMF mitochondria perimeter but an increase of the same parameter with respect to SS mitochondria. The reason for this differential effect is unclear and has not been addressed yet. IMF mitochondria play the dominant role in supplying ATP for muscle contraction and thus must undergo constant modulation of their number and morphology through the processes of fusion and fission to optimize their metabolic function. These considerations may explain why they are particularly susceptible to modification of their microenvironment, such as through the production of ROS, compared to SS mitochondria [28].

Although P110 treatment significantly reduced aberrant mitochondrial dynamics, limited ROS production, and prevented VIDD, it has few effects on mitochondrial perimeter and the number of Z-lines spanned by mitochondria. These results suggest that other proteins involved in the fission process such as dynamin 2, mitochondrial fission factor, and cardiolipin (the latter two have interactions with DRP1 and respective roles in the fission process that are poorly known) are also important to take into consideration when reversing aberrant mitochondrial dynamics induced by MV.

Mitochondrial function and structure are intimately linked. Previous studies have demonstrated that reducing mitochondrial fragmentation by modulating the fission/fusion balance is sufficient to restore mitochondrial function [17, 29]. In line with our results, inhibition of mitochondrial fission by down-regulating FIS1 and DRP1 prevented fragmentation as well as conferred resistance to mitochondrial depolarization and cell death when exposed to a variety of exogenous apoptotic inducers such as oxidative stress [12, 30].

We previously reported oxidative damage to other components of the contractile machinery of the diaphragm during VIDD including RyR1 Ca^2+^ channels [20]. RyR1 oxidation had led to intracellular Ca^2+^ leak and diaphragm weakness prior to diaphragmatic injury and atrophy. Here, we show, for the first time, that P110 reduced ROS production and prevented RyR1 oxidation, nitrosylation, and the subsequent diaphragm weakness induced by 6 h of MV in mice. Thus, mitochondrial fission and ROS production are likely upstream signaling of Ca^2+^ dyshomeostasis in VIDD. Indeed, a growing body of evidence indicates that RyR1 is tightly regulated by posttranslational modifications involving remodeling of the RyR1 macromolecular complex [31] which contain over 20 exposed cysteine residues susceptible to oxidation. These modifications have functional consequences on the Ca^2+^ release channels and muscle function [32]. As previously reported [19, 20], we have seen increased RyR1 remodeling within 6 h of MV and calstabin1 dissociation in mice, suggesting that the increase of mROS production linked to mitochondrial fragmentation induced by MV is a major contributing factor to RyR1 oxidation and calstabin1 depletion. Thus, P110 treatment could be a promising therapeutic intervention at an early stage of VIDD onset. Of note, our proteomic analysis showed down-regulation of several sarcomeric protein levels including tropomyosin 1, 2, and 3, myosin heavy chain 7, and troponin I3. Whether this early sarcomeric protein dysregulation is mediated by oxidative stress and RyR1 Ca^2+^ leak remains to be investigated in future studies.

The inhibitory peptide P110 comprises a 7-amino acid sequence that shares homology between DRP1 and FIS1 [33]. In a clinical perspective, it is important to consider that P110 does not affect DRP1 interactions with any other mitochondrial adaptor of DRP1 nor with the mitochondrial fusion proteins, MFN1 or MFN2 [17, 33]. P110 specifically inhibits excessive mitochondrial fission while preserving the basal level, thus enabling the normal process of mitophagy to take place [17].

Indeed, administering P110 to wild-type mice for a period of five months is well-tolerated and does not impact mitochondrial structure or function [34].

Cells stress markers, bodily metabolism, and behavior were also not affected [34]. Moreover, in the present study, we did not notice any effect on the expression of other mitochondrial shaping proteins after P110 treatment. These data further confirm the conclusions of previous preclinical studies indicating that mitochondrial oxidative stress is an essential element of VIDD and a potential therapeutic target [2, 11].

Although the primary cause of mitochondrial oxidative stress remains unclear [35], we suggest that mitochondrial morphology alterations due to the MV are a requirement for mitochondrial ROS production and VIDD. Nevertheless, the upstream signal of mitochondrial fission remains unknown. We previously demonstrated that prolonged MV in rabbits was associated with disrupted myofibrils, smaller mitochondria with focal membrane disruptions, and a decreased efficiency of oxidative phosphorylation coupling [36]. Here, at an early stage of MV, the structural anomalies observed seem to be limited to the mitochondria. Our present study suggests, in accordance with a previous study [2], that the reduced energy requirement associated with mechanical diaphragm inactivity at early stages of MV might be interpreted by the muscle fibers as a signal to down-regulate mitochondrial activity and enzymatic function through the maintenance of a steady adenosine monophosphate (AMP)/ATP ratio. This would promote a rapid transition to mitochondria fragmentation to decrease the pool of available mitochondria able to produce ATP. Indeed, a recent study has shown, in mechanically ventilated patients, decreased AMP-activated protein kinase and Sirtuin expression, reduction of mitochondrial biogenesis transcription factor expression, and presence of ectopic lipid within muscle fibers suggesting a high metabolic oversupply [2].

### Limits of our findings

In the present study, we only focused on two posttranslational modifications of RyR1 which contributed to the SR Ca^2+^ leak to explain the subsequent muscle weakness induced by MV. However, our results on the early effects of MV on diaphragmatic proteomics also show early deregulation of cellular pathways that converge toward a deep mitochondrial alterations, Ca^2+^ dyshomeostasis, and activation of the JAK-STAT pathway. JAK-STAT and Smad3 pathways might be the signals linking mechanical inactivity to mitochondrial abnormalities. This pathway is stimulated early during MV [4, 21], and we previously reported that it could be considered as an upstream signaling pathway of mitochondrial oxidative stress and RyR1 remodeling [20]. Consistent with this hypothesis, muscle and fibroblasts of patients with STAT2 deficiency or mutation or SHSY5Y-STAT2 knockdown cells exhibited extremely elongated mitochondria, related to a fission process deficit [37]. Interestingly, in these knockdown cells, DRP1 phosphorylation at serine 616 (known to activate DRP1) was significantly reduced and such phenotype was rescued by STAT2 wild-type lentiviral transduction.

Finally, Den Berg et al. have reported diaphragm weakness and atrophy with preserved mitochondrial function and morphology in ventilated critically patients [38]. This is not consistent with our results and with previous studies in terms of mitochondrial abnormalities [2, 39]. One explanation that may account for this discrepancy is the preserved level of diaphragm contractile activity during MV in the patients included in their study, which may protect them against mitochondrial dysfunction [40]. This was not the case in animal models, which are ventilated slightly under hypercapnia to inhibit central respiratory drive and diaphragm contractile activity.

## Conclusion

**Fig. 7.**
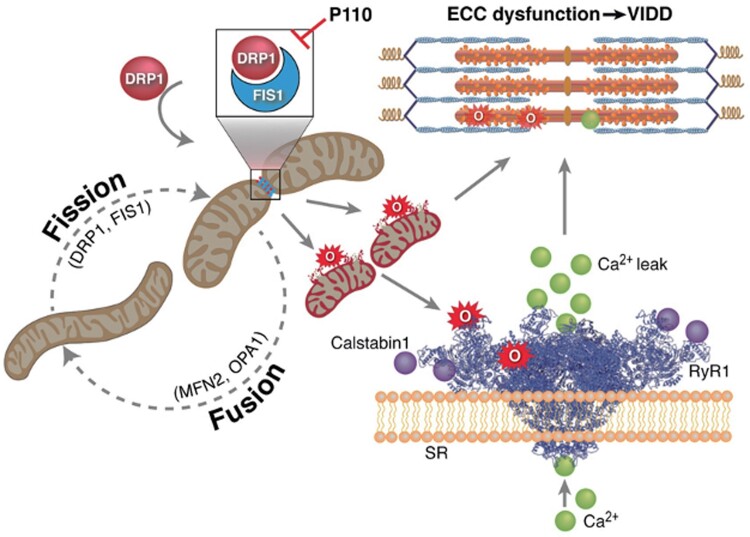
Schematic representation of mitochondrial dynamics imbalance involved in ventilator-induced diaphragmatic dysfunction. Diaphragm unloading during MV leads to mitochondrial fragmentation. Mitochondrial fission is primarily mediated by DRP1. Once recruited to outer mitochondrial membrane, DRP1 binds to its specific receptor FIS1, forms a multimeric ring around the mitochondria, and splits it into two small organelles. This process is termed mitochondrial fragmentation or fission. Excessive mitochondrial fission increases mitochondrial ROS production which oxidizes the calcium release channel type 1 Ryanodine receptor (RyR1) and sarcomeric proteins [4]. RyR1 oxidation dissociate the calcium stabilizing binding protein (calstabin1) and leads to unstable and leaky channels. RyR1 calcium leak alters excitation–contraction coupling and leads to diaphragm weakness associated with MV. P110 inhibitor binds to DRP1 receptor “FIS1” and reduces mitochondrial fission, mitochondrial ROS production, RyR1 oxidation as well as SR calcium leak and thereby prevents diaphragm dysfunction induced by MV. Drp1: dynamin-related protein 1; FIS1: mitochondrial fission 1 protein; MFN2: Mitofusin 2; OPA1: mitochondrial dynamin-like GTPase; SR: sarcoplasmic reticulum; RyR1: ryanodine receptor type 1; ECC: excitation–contraction coupling; VIDD: ventilator-induced diaphragmatic dysfunction.

The inactivity of the diaphragm induced by MV is an important signal that induces proteomic alterations which mainly affect the mitochondria and trigger its fission to meet the sudden decrease of metabolic demand. In this study, we observed that 6 h of MV in mice induces excessive mitochondrial fragmentation leading to mROS production, which is involved in a rapid remodeling of the RyR1 in the diaphragm and subsequent VIDD. These results are summarized in Fig. 7. To the best of our knowledge, this study represents the first evidence of mitochondrial fragmentation as a proximal mechanism underlying VIDD and the binding of DRP1 to its mitochondrial membrane receptor as a potential target for therapeutic intervention.

## Methods

### Experiments on animals and institutional approval

The study adhered to the animal experiment protocols outlined by the institutional animal care committee in Montpellier, France, in accordance with the recommendations of the World Medical Association, documented under reference #CEEA-LR-12078. Healthy male C57/BL6 mice (aged 10–12 weeks and weighing 25–30 g) were acclimatized in our animal facility for one week before initiating the experiments.

### Experimental design

As previously described in our earlier study [20], mice were randomly assigned to one of three experimental groups: (1) mice mechanically ventilated for 6 h (MV, *n* = 8), (2) mice mechanically ventilated for 6 h and treated by P110 (MV + P110, *n* = 8), and (3) control (*n* = 8). P110 molecule was dissolved in Ringer's lactate (RL) solution and delivered in a bolus (loading) dose ((3 mg/kg) intraperitoneal (IP) injection) 1 h before initiation of MV. A constant IP injection (3 mg/kg/h) of P110 was maintained throughout MV.

RL was used as vehicle for MV group. Airway pressure and hemodynamic parameters were monitored and maintained within the physiological range as previously reported [41]. P110 is a heptapeptide conjugated to TAT47–57 that specifically blocks the interaction between DRP1 and FIS1, an adaptor protein located on the mitochondria [18].

### Chemical details of P110: a pharmacological inhibitor of DRP1 receptor

The cell permeable peptide, TAT-P110 (Tyr-Gly-Arg-Lys-Lys-Arg-Arg-Gln-Arg-Arg-Arg-Gly-Gly-Asp-Leu-Leu-Pro-Arg-Gly-Thr), molecular weight = 2426.7 g/mole, was synthesized by Genemed Synthesis Inc. (San Antonio, TX) to >99% purity as determined by high-performance liquid chromatography.

### MV

Mice in the MV groups underwent anesthesia through an IP injection of pentobarbital sodium (50 mg/kg body weight) and were orally intubated using a 22-gauge angio-catheter. No neuromuscular blocking agents were administered. MV was conducted using a small animal ventilator (Minivent, Harvard Apparatus, Saint-Laurent, Quebec, Canada) with the following settings: a fraction of inspired oxygen of 0.21 (room air), controlled volume mode with a tidal volume of 10 µl/mg body weight, a respiratory rate of 150 breaths/min, and positive end-expiratory pressure maintained at 3–4 cm H_2_O by placing the expiratory port under a water seal. General care for the MV groups included continuous maintenance of body temperature using a homeothermic blanket (Homeothermic Blanket Control unit; Harvard Apparatus, Saint-Laurent, Quebec, Canada), and hourly IP injections of 0.05 ml of ringer lactate solution were administered to maintain hemodynamic stability and compensate for insensible losses. Additionally, bladder expression, ocular lubrication, and passive limb movements were performed as part of the care protocol. Anesthesia was sustained by IP injection of pentobarbital sodium (5 mg/kg body weight/hour) throughout the 6-h duration of MV.

### Transmission electron microscopy

For transmission electron microscopy (TEM) analysis, diaphragm muscle strips (5 × 15 mm) were fixed in a 2% glutaraldehyde solution in 0.1 M cacodylate buffer (pH 7.4). The samples underwent a series of processing steps. Initially, they were fixed with 1% osmium tetroxide and then dehydrated using acetone. Following dehydration, the samples were impregnated with resin. Smaller portions of diaphragm tissues were subsequently embedded in 100% resin either in cross-sectional (transverse) or longitudinal orientations. To ensure the proper orientation and quality of sections, 1-μm-thick sections were cut and stained with a solution containing 1% toluidine blue and 1% borax. Ultrathin sections measuring 70 nm were then sliced using a diamond knife on a Leica ultramicrotome (Leica-Reichert Ultracut E). These sections were stretched with chloroform vapor and mounted on copper grids coated with Pioloform film before being stained with 2% aqueous uranyl acetate and lead citrate.

Subsequently, ultrathin sections were examined using a Tecnai F20 transmission electron microscope at 200 kV, located in the CoMET Montpellier Rio Imaging facilities at the Institute for Neurosciences of Montpellier, France. Digital micrographs were captured from each sample at magnifications of 13,500× and 19,000×. Tissues from four mice per group were collected, imaged, and analyzed for both cross-sectional and longitudinal orientations.

### Morphological analysis

For each animal, randomly selected mitochondria-rich oxidative muscle fibers were analyzed in both transverse and longitudinal sections as previously described in detail [42]. In short, for the analysis of intramyofibrillar (IMF) mitochondrial morphology, muscle samples were cut in a transverse (cross-sectional) orientation. To achieve the most precise transverse orientation at the subcellular level, the muscle fibers were sliced in a nearly parallel orientation to the Z-line.

In total, an observer who was unaware of group assignments analyzed 2,349 IMF mitochondria from the control group, 1,494 IMF mitochondria from the MV group, and 2,099 IMF mitochondria from the MV + P110 group. The assessment of SS mitochondrial morphology was conducted at a magnification of 19,000× in the longitudinal orientation.

Mitochondrial perimeters were determined using Image J software (version 1.41; National Institutes of Health, Bethesda, MD) by manually delineating the borders of intramyofibrillar (IMF) and SS mitochondria, which exhibited well-defined outlines in TEM micrographs.

To assess interactions between intramyofibrillar (IMF) and SS mitochondria, we examined micrographs of longitudinally aligned myofilaments obtained from four mice per group. They were captured at magnifications of 13,500× for IMF mitochondria and 19,000× for SS mitochondria.

In the IMF compartment, a total of 689 (control), 648 (MV), and 534 (MV + P110) Z-lines possessing mitochondria on both sides, either as pairs of discrete organelles, as a continuous mitochondrion, or as interacting organelles joined by an electron-dense IMJ, were analyzed. The proportion of Z-lines spanned by continuous or interacting mitochondria is reported as a percentage of the total number of Z-lines analyzed. In addition, the proportion of electron-dense physical contact sites between adjacent mitochondria was determined in all the groups in both orientations as described previously [43].

### Biochemical evaluation: protein extraction

Proteins were extracted from frozen costal diaphragms (20 mg) homogenized with a manual polytron^®^ instrument. Tissues were then lysed in 600 µl extraction buffer (Tris maleate 10 mM, NaF 35 mM, Triton 1%, activated orthovanadate 20 mM, protease inhibitor cocktail (Roche, France)), and agitated for 45 min by rotation. Membrane and cytosolic proteins were collected from the supernatant after 5 min of centrifugation at 10,000*×g* at 4 °C.

### SS and IMF mitochondria isolation and ROS production measurement

Diaphragms were minced and homogenized in 1 ml of homogenate buffer (sucrose 0.3 M, TES 5 mM, ethylene glycol-bis(β-aminoethyl ether)-N,N,N′,N′-tetraacetic acid (EGTA) 0.2 mM, pH 7.2) with a polytron homogenizer (15,000 rpm, 60 s). The homogenate was centrifuged at 1,000*g* for 10 min at 4 °C. The mitochondria pellet was suspended in 4 ml of Bovine Serum Albumin (BSA) buffer (sucrose 0.3 M, TES 5 mM, EGTA 0.2 mM, BSA 1 mg/ml, pH 7.2) and nargase (5 mg/g) (Sigma 9014-01-1) was added and centrifuged at 12,000*g* for 15 min at 4 °C. The new pellet was resuspended in 2 ml of BSA buffer and centrifuged at 1000*g* for 10 min at 4 °C. The supernatant was then centrifuged at 12,000*g* for 15 min at 4 °C and the pellet containing IMF mitochondria was resuspended in 100 μl of homogenate buffer and used for mitochondrial ROS production assay. The supernatant obtained at the first step was centrifuged at 12,000*g* for 15 min at 4 °C and the pellet containing SS mitochondria was resuspended in 100 μl of homogenate buffer and used to measure the SS mitochondrial ROS production.

Mitochondrial ROS production was measured using the 2′7′-dichlorofluorescein (DCF)-diacetate (DA) method. 50 μg of SS or IMF mitochondria were incubated at 37 °C for 1 h with 20 μM H2DCF-DA (Thermo Fisher Scientific), a compound that becomes highly fluorescent as a result of activation by ROS, producing a fluorescent molecule known as DCF. The fluorescence emitted by DCF was then recorded at 490/528 nm for a duration of 80 min using a multimode plate reader.

### Mitochondrial Ca^2+^ content

Isolated mitochondria were suspended in homogenate buffer (sucrose 0.3 M, TES 5 mM, pH 7.2) without EGTA, and an aliquot of this suspension was taken to determine protein concentration by a bicinchoninic acid assay kit. The mitochondria were washed with 1 ml of homogenate buffer (without EGTA) and then centrifuged at 16,000*g* for 5 min. The pelleted mitochondria were resuspended in HCl 0.6 N, homogenized, and sonicated. The mitochondria were then incubated in boiling water. The mitochondria lysates were then centrifuged for 5 min at 10,000*g*, the supernatant was recovered, and its pH was adjusted to between 6 and 8. Ca^2+^ content in the supernatant was determined spectrophotometrically (Tecan) using an o-cresolphthalein Complexone assay kit (Cayman Chemical). Results were expressed in nanomoles of Ca^2+^ per milligram of protein.

### Mitochondrial respiration

The mice were euthanized by cervical dislocation. The costal diaphragm was surgically removed. The diaphragm was gently minced with a scissor for 2 min and then weighed. Four volumes of ATP buffer (100 mM KCl, 5 mM MgSO_4_, 5 mM ethylenediaminetetraacetic acid), and 50 mM Tris-HCl) and the protease subtilisin A (0.1 mg/g of tissue) were added to the mince, and the solution was incubated for 5 min in ice. Reaction was then stopped with 10 volumes of ATP buffer and the solution was homogenized with a polytron homogenizer (15 s at 40% of full power). The homogenate was centrifuged at 8,500*g* for 10 min at 4 °C, supernatant was removed, and the pellet resuspended with 10 volumes of ATP buffer and again centrifuged at 800*g* for 10 min at 4 °C. The supernatant obtained was recovered and centrifuged for 10 min at 9,000*g* at 4 °C. Once again, the supernatant was removed, and the pellet was resuspended with 3.5 ml of ATP buffer and centrifuged at 9,000*g* for 10 min at 4 °C. Finally, supernatant was eliminated, and mitochondria were resuspended in 30 µl of resuspension buffer (100 mM KCl, 10 mM 3-(N-morpholino)propanesulfonic acid, pH 7.4). The mitochondrial protein content was assessed using the Bradford assay, and the results were quantified as milligrams of mitochondrial proteins per gram of muscle wet weight.

Mitochondria oxygen consumption was measured using the high-resolution Oxygraph-2k (ORO-BOROS Instruments, Innsbruck, Austria). Mitochondria were placed inside two sealed, temperature-controlled chambers (37 °C) containing 2 ml of MIRO5 respiration medium (0.5 mM EGTA, 3 mM MgCl_2_·6H_2_O, 65 mM KCl, 20 mM taurine, 10 mM KH_2_PO_4_, 20 mM 4-(2-hydroxyethyl)-1-piperazineethanesulfonic acid (HEPES), 110 mM sucrose, and 1 g/l BSA, pH 7.1 [10]).

The resting rate (state 4) was assessed when mitochondria were exposed to a solution containing 2.5 mM malate and 5 mM pyruvate. The adenosine diphosphate (ADP)-stimulated rate (state 3) was measured following the addition of 0.25 mM ADP.

It represents the recovery rate after completion of ATP synthesis (defined as oxygen consumption of mitochondria after the depletion of exogenous ADP). The acquisition and analysis of data were conducted utilizing Oxygraph-2k-DatLab software version 4.3, provided by ORO-BOROS Instruments. The RCR was defined as the ratio of oxygen consumption during state 3 to oxygen consumption during state 4.

For quality control purposes, control diaphragms were always assessed along the ventilated or the ventilated and treated diaphragms at the same day and under the same condition and instrument calibration.

### DRP1 recruitment to the mitochondria

Isolated IMF and SS mitochondria were mixed with sample buffer and boiled for 5 min before gel loading. Mitochondrial proteins were transferred onto nitrocellulose membranes for 2 h at 200 mA. Immunoblots were developed using anti-DRP1 antibodies: (ab 184247, Abcam).

### RyR1 immunoprecipitation

As described in our previous study [20], RyR1 was immunoprecipitated from diaphragm biopsies with 2μg of RyR1 specific antibody “5029” [44] in 0.5 ml of a modified radioimmune precipitation assay buffer (50 mm Tris-HCl, pH 7.2, 0.9% NaCl, 5.0 mm NaF, 1.0 mm Na_3_VO_4_, 1% Triton X-100, and protease inhibitors (Roche)) overnight at 4 °C.

RyR1-1327 is a rabbit polyclonal antibody that was purified for its binding affinity against a keyhole limpet haemocyanin-conjugated peptide with the amino acid sequence CAEPDTDYENLRRS. The sequence corresponds to amino acid residues 1,327–1,339 of the mouse skeletal RyR1. An additional cysteine residue was added to the amino terminus, and the antibody was subsequently purified using the unconjugated form of the peptide.

As outlined in the methodology section of our prior study [45], the immune complexes were incubated with protein A-Sepharose beads (Sigma) at 4 °C for 1 h, and the beads were washed three times with radioimmune precipitation assay buffer. The immunoprecipitates were size-fractionated on sodium dodecyl-sulfate polyacrylamide gel electrophoresis gels (4–20% for RyR1 and calstabin) and transferred onto nitrocellulose membranes for 2 h at 200 mA. Immunoblots were developed using the following primary antibodies: anti-RyR1 (Affinity Bioreagents; 1:2,000), anti-cysteine nitrosylation (anti-Cys-NO) antibody (Sigma-Aldrich, St. Louis, MS, USA; 1:2,000), anti-calstabin (FKBP12 C-19, 1:1,000; Santa Cruz Biotechnology, Inc., Santa Cruz, CA), and anti-4 hydroxynonenal (Abcam; 1:1,000). To determine the extent of channel oxidation, the carbonyl groups in the protein side chains were derivatized to 2,4-dinitrophenylhydrazone (DNP) by reaction with 2,4-dinitrophenylhydrazine. The DNP signal associated with RyR was determined using a specific anti-DNP antibody according to the manufacture instructions (Millipore, Billerica, MA). All immunoblots were developed with the Odyssey system (LI-COR Biosciences, Lincoln, NE) using infrared-labeled anti-mouse and anti-rabbit IgG (1:10,000 dilution) secondary antibodies as previously described [19, 20].

### Mitochondrial shaping proteins analysis

For mitochondrial shaping proteins analyses, muscle lysates extracted from diaphragms were combined with sample buffer and subjected to boiling for 5 min prior to loading onto the gel. The concentrations of primary and secondary antibodies were prepared in accordance with the manufacturers' recommendations. The quantification of distinct protein bands was carried out using the Odyssey Infrared Imaging System (LI-COR Biosciences, Lincoln, NE), with glyceraldehyde-3-phosphate dehydrogenase serving as a loading control to normalize for potential variations in total protein loading.

The following proteins were detected: MFN2 (ab 56889; Abcam), PGC1-α (ab 54481; Abcam), and OPA1 (611112; BD Biosciences).

### Measurement of diaphragm contractile properties

The mice were euthanized by cervical dislocation. The costal diaphragm was surgically removed to permit measurements of isometric contractile properties as described in our previous study [7]. The muscle was extended to L0, which represents the length at which the muscle generates its highest isometric tension, and subsequently was subjected to supramaximal stimulation using square wave pulses (delivered via Model S48; Grass Instruments, West Warwick, RI). The force–frequency relationship was established by consecutively stimulating the muscles at frequencies of 10, 20, 30, 50, 60, 80, 100, and 120 Hz, each lasting for 600 ms, with 1-min intervals between each stimulation train.

After completion of the measurements of contractile properties, muscle was measured for its length, dried for 5 min to remove the surface buffer, and weighed. The muscle's cross-sectional area was calculated by dividing its weight by the product of its length and tissue density (1.056 g/cm³). Subsequently, diaphragmatic force production was adjusted by normalizing it to the muscle's cross-sectional area, resulting in specific force measurements expressed in Newtons per square centimeter (N/cm²).

### SR vesicle preparation

Using the same methodology we described previously [19], diaphragm's biopsies were homogenized on ice in 300 mM sucrose, 20 mM Pipes (pH 7.0) in the presence of protease inhibitors (Roche), and centrifuged at 8,000 rpm (5,900*×g*) for 20 min at 4 °C. The supernatant was ultracentrifuged at 32,000 rpm (100,000*×g*) for 1 h at 4 °C. The final pellet containing microsomal fractions enriched in SR vesicles was resuspended and aliquoted in 300 mM sucrose, 5 mM Pipes (pH 7.0) containing protease inhibitors. Samples were frozen in liquid nitrogen and stored at −80 °C.

### Measurement of the open probability of RyR1

Employing the methodology we previously detailed [45], planar lipid bilayers were formed using a 3:1 mixture of phosphatidylethanolamine and phosphatidylcholine (Avanti Polar Lipids) suspended (30 mg/ml) in decane by painting the lipid/decane solution across a 200-µm aperture in a polysulfonate cup (Warner Instruments) separating two chambers. The trans chamber (1 ml) representing the intra-endoplasmic reticulum/SR (luminal) compartment was connected to the head stage input of a bilayer voltage clamp amplifier (BC-525D, Warner Instruments) and the cis chamber (1 ml), representing the cytoplasmic compartment, was held at virtual ground. Solutions in both chambers were as follows: 1 mM EGTA, 250/125 mM Hepes/Tris, 50 mM KCl, 0.64 mM CaCl_2_, pH 7.35 as cis solution and 53 mM Ca(OH)_2_, 50 mM KCl, 250 mM Hepes, pH 7.35 as trans solution [19].

The concentration of free Ca^2+^ in the cis chamber was calculated using the WinMaxC program (version 2.50; www.stanford.edu/∼cpatton/maxc.html). SR vesicles were added to the cis side, and fusion with the lipid bilayer was induced by making the cis side hyperosmotic through the addition of 400–500 mM KCl. After the appearance of potassium and chloride channels, the cis compartment was perfused with the cis solution. Single-channel currents were recorded at 0 mV by using a Bilayer Clamp BC-535 amplifier (Warner Instruments), filtered at 1 kHz and digitized at 4 kHz. All experiments were performed at room temperature. Data acquisition was performed using Digidata 1440A and Axoscope 10.2 software, and recordings were analyzed using Clampfit 10.2 (Molecular Devices). Open probability was identified by 50% threshold analyses using a minimum of 2 min of continuous record. At the conclusion of each experiment, ryanodine (5 µM) was added to the cis chamber to confirm channels as RyRs.

### Global quantitative proteomics analysis

For global quantitative proteomics of fresh frozen diaphragms from controls and MV mice, diaPASEF [46] (data independent acquisition) based proteomics was used. Tissues were lysed by bead-beating in lysis buffer [47] (2% SDS, 1% sodium deoxycholate (SDC), 100 mM Tris-HCl pH 8.5, and protease inhibitors) and boiled for 10 min at 95 °C, 1,500 rpm. Protein reduction and alkylation of cysteines was performed with 10 mM tris(2-carboxyethyl)phosphine and 40 mM 2-chloroacetamide at 45 °C for 10 min, followed by sonication in a water bath and cooling down to room temperature. Cleared lysate was precipitated with the acetone-salt method, as previously described [48], and precipitated pellets were resuspended in SDC buffer (1% SDC and 100 mM Tris-HCl pH 8.5).

The protein digestion process involved an overnight incubation, where LysC and trypsin were added at a ratio of 1:50 (enzyme to protein, µg to µg) and maintained at 37 °C with continuous shaking at 1,400 rpm. Subsequently, peptides were acidified by introducing 1% trifluoroacetic acid (TFA), followed by vortexing and purification via StageTip clean-up using styrenedivinylbenzene RPS. The peptides were loaded onto a StageTip plug with a 14-gauge needle and subjected to two washes with 200 µl of 1% TFA/99% ethyl acetate and 200 µl of 0.2% TFA/5% acetonitrile (ACN), respectively, in a centrifuge at 3,000 rpm. Afterward, elution was performed using 60 µl of a solution containing 1% ammonia and 50% ACN into Eppendorf tubes, followed by drying at 45 °C in a SpeedVac centrifuge. Samples were then resuspended in 10 μl of liquid chromatography buffer (3% ACN/0.1% formic acid), and the peptide concentrations were determined using NanoDrop. For subsequent Parallel Accumulation Serial Fra gmentation (PASEF) and diaPASEF analysis on the timsTOFPro instrument, 200 ng of each sample was utilized.

For spectral library generation, 10 μg of each digested tissue sample was pooled and dried in a speedVac. Pooled dried peptides were resuspended in 100 μl of 1% TFA, pH 2 and subjected to fractionation with mixed mode styrenedivinylbenzene SCX StageTip [48]. Peptides were fractionated into nine fractions; each fractionated peptide was dissolved in 10 µl of 3% ACN/0.1% formic acid and injected using the PASEF method.

Peptides were separated over a period of 120 min at a flow rate of 400 nl/min. This separation was performed on a reversed-phase C18 column equipped with an integrated CaptiveSpray Emitter (25 cm × 75 µm, 1.6 µm, IonOpticks). The mobile phases consisted of A, which was 0.1% formic acid in water, and B, which was 0.1% formic acid in ACN. The fraction of mobile phase B was increased linearly from 2 to 23% over 90 min, followed by a 10-min gradient to 35%, and finally, an increase to 80% before re-equilibration. The timsTOF Pro instrument operated in PASEF mode with specific settings as follows:

Mass range 100–1,700 *m*/*z*, 1/K0 start 0.6 V s/cm^2^, end 1.6 V s/cm^2^, ramp time 100 ms, lock duty cycle to 100%, capillary voltage 1,600 V, dry gas 3 l/min, dry temp 200 °C, PASEF settings: 10 Tandem Mass Spectrometry (MSMS) frames (1.16 s duty cycle), charge range 0–5, active exclusion for 0.4 min, target intensity 20,000, intensity threshold 2,500, and Collision-induced dissociation energy 59 eV. We applied a polygonal filter in both the *m*/*z* and ion mobility dimensions to isolate features that were more likely to correspond to peptide precursors rather than singly charged background ions.

DiaPASEF [46] experiment was acquired at defined 32 × 25 Th isolation windows from *m*/*z* 400–1,200. In order to customize the MS1 cycle time for diaPASEF, we configured the repetitions as follows: two repetitions were set for the 16-scan diaPASEF scheme, and four repetitions were established for the 4-scan diaPASEF scheme in these experiments.

The collision energy was ramped linearly as a function of the mobility from 59 eV at 1/K0 = 1.6 V s cm^−2^ to 20 eV at 1/K0 = 0.6 V s cm^−2^. To construct sample-specific spectral libraries, we conducted searches on the acquired PASEF raw files and diaPASEF raw files against the UniProt mouse database using the Pulsar search engine. This was accomplished by utilizing the hybrid spectral library generation feature of Spectromine with default settings [49].

The raw protein intensities were determined by adding together the intensities of the constituent peptides.

DiaPASEF data were analyzed with Spectronaut Pulsar X [49], a mass spectrometer vendor software independent from Biognosys. We employed the default settings for the targeted analysis of diaPASEF data in Spectronaut, with the exception of configuring the decoy generation to be mutated.

We estimated the FDR using the mProphet approach, aiming to establish it at 1% at both the peptide precursor and protein levels. The data generated from Spectronaut underwent additional analysis utilizing the Spectronaut statistical package.

The threshold criteria for identifying differentially expressed proteins comprised a *P*-value < 0.05, corrected through permutation-based FDR, a fold-change ≥ 1.5, and unique peptides ≥ 2.

The significantly changed proteins between controls and MV diaphragms were used for volcano plot, heatmap, GO, and KEGG analysis. We also conducted GSEA to pinpoint the statistically significant gene sets within a ranked list of genes.

### Statistical analysis

Data are presented as mean values with the standard error of the mean (SEM). Statistical significance was defined as *P* < 0.05, determined through Student's unpaired *t*-test or analysis of variance (one-way or two-way), with subsequent Bonferroni-selected comparison tests.

## Supplementary Material

pgad336_Supplementary_Data

## Data Availability

All data are included in the manuscript and/or supporting information.
