# Supplementary material for: Aberrant mitochondrial dynamics contributes to diaphragmatic weakness induced by mechanical ventilation
Source: PNAS Nexus. 2023 Nov 7;2(11):pgad336. doi: 10.1093/pnasnexus/pgad336 (PMC10635656; doi:10.1093/pnasnexus/pgad336)
Supplement: pgad336_Supplementary_Data [file pgad336_supplementary_data.zip › PNASNEXUS-PNASNEXUS-2023-00277RR-s02.pdf]

Supplementary table 1

Log2 Ratio	padj	Gene
-0,67258169	1,72E-05	Abcf2
-1,099972893	0,000154584	Abcg2
0,602775927	0,023516499	Acaca
0,581304613	0,026483912	Acly
-0,797226759	0,024814799	Acot11
-1,207137025	2,22E-06	Adal
-0,716480583	0,00116968	Adamts12
-0,888629955	1,34E-06	Aebp1
-0,972888187	3,58E-06	Ago3
-1,270486917	0,000996853	Ahsp
-0,774098956	7,99E-08	Akap5
-0,813858503	0,001880058	Aldh3a2
0,779523896	3,40E-06	Alpk3
-0,596626892	0,000257049	Alpl
-0,589300467	0,005677508	Ampd3
-1,34403756	0,00054636	Amph
-0,777800763	0,001418536	Ank1
-0,887879456	6,05E-06	Anp32b
-0,737357932	0,000549429	Anxa9
-3,046819005	0,000903625	Apoc1
-1,115534987	0,001235285	Apoc3
-0,78040397	1,95E-05	Arsb
-0,914025522	4,78E-05	Atox1
-0,810524588	9,05E-05	Atp6v1g2
-0,686500527	6,90E-06	Banf1
1,1436699	0,000347932	Bckdk
-0,888730298	2,77E-05	Bnip1
-0,614937281	0,038569102	Bpgm
-0,994833788	2,66E-06	Bpifa2
-1,161408447	0,00640501	Brap
0,710552272	0,001170318	C1qa
-0,690592765	0,015769078	Ca1
-0,726433426	0,002071858	Cab39l
-0,625254112	0,00057295	Camk2a
2,834070326	0,000534223	Camp
-1,616636173	1,13E-06	Ccar1
0,643833839	3,95E-06	Ccny
0,700841529	0,01781075	Cdh1
0,82169103	0,000953329	Cdk9
-1,262224074	0,003555457	Cela1
-1,299234243	0,000536499	Cela2a
1,339811554	0,005030286	Cela3b
-0,681175727	7,20E-05	Cenpv
0,739315341	0,00713979	Ces1f
-0,715599374	6,50E-08	Cfdp1
0,743873021	0,006635612	Chi3l1
2,192211945	0,000372249	Chil3
-0,662488083	0,00081633	Chmp6
1,27663548	2,34E-06	Ciao1

-0,922563722 0,000201528 Cirbp
-1,321760828 9,12E-05 Cmc1
0,895853139 6,24E-06 Cmya5
-0,763662487 0,006941501 Col12a1
-0,600277259 0,03934554 Col5a2
0,658877892 0,00102566 Col6a5
0,704473118 2,49E-06 Coq5
-0,589894798 0,001044608 Cox15
-0,729065604 0,000525616 Cox17
-0,610543641 0,011644389 Cox6b1
-0,8958322 0,011140428 Cox7a2
-2,204440513 0,003511706 Cox7c
0,642798399 0,004217666 Cryzl2
-0,842973144 0,001210374 Cth
-0,937425771 1,03E-05 Ctnnd1
0,75211834 0,004366508 Ctss
-0,592456765 9,69E-05 Cuedc2
1,1740111 0,00065428 Cybb
-0,731033933 1,07E-06 Cyp2b19
-1,242489178 9,86E-05 Dad1
-0,745781322 0,000177982 Dcaf7
-0,627123939 7,59E-05 Dctn3
-0,599151352 0,00073831 Dkc1
0,896199373 1,32E-11 Dmd
-0,967064844 0,00069123 Dmtn
-1,356216971 3,65E-05 Dnajc19
-1,280779894 6,90E-06 Dph1
-0,873238506 0,000325062 Dpt
-0,936165387 0,02275475 Dpys
-0,726176261 0,000222028 Dsp
0,798212026 6,09E-06 Dst
-1,871289351 0,009794349 Dtd2
0,596594574 0,025738676 Dusp12
-0,582368077 0,004086348 Ecm1
-0,769916667 5,12E-08 Edf1
-0,871250021 3,74E-05 Eif4ebp1
1,969577077 0,000754237 Elane
-0,99808237 0,000515063 Elmo1
-0,606771199 3,12E-05 Emd
-0,713630044 0,000448898 Ensa
0,86963208 2,72E-05 Epb41l1
-1,181256119 1,48E-05 Espn
-1,630113883 1,72E-05 Ewsr1
-0,682983531 0,000261503 Fam234a
2,248253718 4,39E-06 Fbxo32
0,605453963 0,029772449 Fbxo44
-0,637311583 2,42E-05 Fmo2
-1,068996626 2,45E-05 Fus
-1,097158679 0,011644389 G6pc3
-1,240966744 3,07E-05 Gga2
-0,72549549 0,000941417 Ghitm
-0,589208455 0,029387618 Gipc1
-0,706526633 0,02937959 Gp1ba
-0,60959481 0,012035647 Gpld1

-0,701451019 0,002206718 Gtf3c1
0,855999967 8,66E-05 Gusb
-1,182113921 0,000104475 H1-0
-0,709457502 4,03E-05 H1-4
-0,988993462 0,000150057 H1-5
-0,676056716 0,006809602 Hba
-0,610355344 0,012335765 Hbb-b1
1,570235526 0,008117759 Hbb-b2
-0,753980114 0,004649905 Hbb-bh1
-0,713614984 0,000182727 Hdgfl3
-0,851493094 4,50E-05 Heatr5a
1,19671386 0,000352547 Hk3
-0,801649992 0,024414128 Hnrnph2
-0,606054438 4,67E-06 Hp
-0,93956361 2,43E-05 Hrg
-0,756881583 0,004925533 Hsd11b1
-0,876682711 2,24E-08 Hspa14
-0,863666287 8,96E-05 Hspb2
0,77875989 0,002937601 Ifit2
-0,709160961 0,006230055 Ighg1
-0,986713342 0,016140905 Ighv3-6
-0,697464356 0,011603194 Ikzf2
-1,337015354 2,17E-06 Irf3
0,78672449 0,002624469 Itgb2
-1,402211435 0,002931143 Jchain
0,65523308 0,00596331 Klhdc10
-1,489612889 9,36E-06 Klhl22
-1,542836785 8,28E-06 Krt75
-0,905406566 0,000280621 Krt8
1,221352243 0,005125127 Lbp
0,662845084 0,001493793 Lbr
2,056486068 0,000196622 Lcn2
0,747270911 0,003977721 Lipe
-1,106093524 0,004768879 Lmnb2
-0,620355998 2,92E-06 Lpin1
-1,066749549 3,80E-06 Lsm3
2,384753336 0,000269635 Ltf
1,199181979 0,000265608 Lyz2
-0,804718107 0,003956616 Macroh2a1
-0,991598995 0,000396045 Maoa
-1,41098457 1,21E-06 Map1lc3a
-0,995270304 9,48E-11 Map1lc3b
-0,653271525 0,000788537 Map4k5
-1,220940165 1,45E-06 Map6
0,795745741 0,012693911 Marcksl1
-0,983912705 6,23E-06 Mecp2
-1,045992006 5,38E-06
"Mef2a,Mef2a,Mef2a;Mef2c,Mef2c,Mef2c,Mef2c,Mef2c;Mef2d,Mef2d"
1,031528091 6,52E-05 Mid1ip1
-0,676734652 4,77E-05 Mif4gd
0,714025707 0,016269166 Mpz
0,806255281 9,03E-05 Mrpl14
0,715319355 4,94E-07 Mt2
-1,811890989 0,005702793 Mtatp8

-1,319157364 0,000470923 Mug2
1,130028434 0,000471009 Mup17
1,046064336 0,001645339 Mup2
1,194350202 0,000117886 Mup20
0,721727334 0,000513518 Mup3
-0,66977393 0,000584903 Myadm
-0,684658131 0,008163297 Mycbp
-0,904645447 0,027119841 Myl9
-1,331474734 1,96E-07 Myoc
-0,851553767 0,005320789 Nadsyn1
-1,016562772 4,62E-06 Nat2
1,001869858 0,000884456 Ncf1
1,374136651 0,000138937 Ncf2
-0,705622807 0,007180099 Ndufa1
-0,591458363 0,012375811 Ndufb8
-1,016797763 6,69E-07 Nedd8
0,590498139 0,027893699 Nefm
0,60912517 0,004060387 Neil2
0,737277313 0,000117109 Nes
-0,625960428 0,005785143 Nexn
2,603826051 0,000396952 Ngp
0,72137524 1,25E-05 Nhej1
0,703918399 6,93E-07 Nme3
-0,980335305 0,000946899 Npl
-0,690543902 1,83E-05 Nt5dc3
-1,785419562 0,000146423 Nudt18
-1,095506856 0,02003149 Numb
-0,877460384 0,003075571 Numbl
0,683792877 0,000243902 Obscn
-1,007803209 1,51E-06 Ocrl
-0,617103863 1,33E-09 Orm2
-2,521758063 1,63E-06 "Oxct2a;Oxct2b"
-0,655572734 3,43E-05 Pacsin1
0,675259359 0,019702971 Pck1
0,60564535 0,000334875 Pdk4
-0,627650159 3,20E-06 Pef1
-0,934388584 9,84E-06 Pfdn1
-0,729235626 1,65E-05 Pfkfb2
0,627523153 0,004808101 Pfn3
0,84254693 0,00637292 Pkia
0,736478959 1,70E-05 Pkp1
-0,884883594 0,000833679 Pla2g12a
0,925397093 0,005561794 Plac8
0,586859834 0,044735023 Pld4
0,761379086 0,020259203 Plin1
-0,923552095 0,000122884 Plpp1
1,105573862 0,004831437 Pmp2
-0,684619024 0,000220164 Pnkd
0,726398601 0,000144469 Pnpla2
-1,096879457 0,002182042 Pnpla8
-0,65507875 0,001990289 Pnpo
-0,769599326 0,002102956 Ppp1r1a
0,689860349 5,10E-05 Ppp1r3a
-0,685290253 0,005081327 Ppp4c

-0,624135353 0,005659966 Proz
1,029617913 0,003770617 Prx
0,732797279 0,000741504 Pstpip2
0,589934736 0,016617993 Ptprc
-0,928706092 5,25E-07 Pxk
-1,408877106 6,43E-05 Pxmp2
-0,735255289 7,02E-05 Pym1
0,638208196 5,98E-05 Raver1
-0,607988355 3,13E-05 Rbbp4
0,87289401 0,00058397 Retnla
2,198147276 0,002358516 Retnlg
0,641216073 0,000520314 Rhbdf1
-0,636880529 5,79E-06 Rpa2
-0,649741975 0,00219154 Rpl13a
-0,845578557 1,06E-05 Rpl22
-0,679030633 0,000511717 Rpl27a
-0,771574811 0,000377813 Rpl30
-0,681789267 0,005414367 Rpl35
-0,719480832 0,000173276 Rplp2
-0,626491034 0,001726527 Rps12
-0,629038087 0,000103821 Rps13
-0,713509839 0,000154469 Rps25
-0,845829494 0,000133933 Rps28
0,636276175 0,00788588 Rrad
-0,733631571 6,34E-05 S100a16
-0,725398357 2,58E-05 S100a6
1,882618694 0,000563706 S100a8
2,61069005 0,000114256 S100a9
-0,929187319 1,83E-05 S100b
-0,591404476 1,32E-11 Saa1
-1,268668655 0,000206922 Saa2
-0,781715537 0,000208463 Saa4
0,811335469 0,002500775 Safb2
-1,021882176 0,00222645 Sat2
-2,167716227 0,033976799 Scamp1
-0,900202813 1,73E-09 Scgb1a1
-0,717015327 7,31E-05 Sco2
-0,605859186 0,000177165 Sdcbp
-0,815963186 0,003474961 Sec11a
-1,074452265 6,48E-05 Selenow
0,608553037 2,29E-05 Skt
-1,280685389 4,06E-05 Slc12a4
-0,802315033 0,000325146 Slc25a13
-0,690549191 0,000570721 Slc25a31
-0,870859719 5,75E-06 Slc35b1
-1,001817385 0,018786454 Slc43a1
-0,630671552 0,000311694 Slfn5
-1,051317651 3,47E-05 Smc1a
-0,832014921 0,000144469 Smg9
-0,85437223 6,00E-05 Smpx
-0,914585059 2,96E-06 Smu1
-1,06979321 0,000154469 Snrnp200
-1,285848213 7,91E-05 Snrpe
-0,650065158 2,10E-07 Snrpg

0,662461699 0,000861011 Sntb1
0,832588247 0,019610602 Sort1
-2,053132875 0,045930217 Spaca1
-1,468142942 0,001172643 Spta1
-0,81681514 1,67E-05 Srp14
-1,007008954 7,54E-07 Srsf2
-0,588570001 0,010232053 Srsf7
-0,720127019 0,035007513 Stfa1
0,611157204 0,008470505 Stfa2
-0,603764043 0,004253943 Stt3a
-1,015665968 0,000504593 Stx1b
-1,090458017 7,15E-05 Sub1
-0,654220638 0,00147707 Sumo2
0,831164725 0,002780495 Surf1
1,090637217 5,12E-08 Svil
-0,581375728 0,00048318 Syn2
0,77219611 9,35E-08 Synm
0,66997543 2,56E-06 Tcp11l2
0,882631416 0,01354015 Tecrl
-1,801284421 6,17E-07 Tefm
-0,790888036 1,09E-06 Tfam
0,92157836 0,046007351 Th
-0,616882167 0,005158377 "Timm8a1;Timm8a2"
0,961178989 0,00034563 Tinagl1
-0,783199599 4,57E-05 Tmed1
-0,980046838 0,001819222 Tmem126b
-0,723962316 0,000272041 Tmem263
-0,862998046 0,000165332 Tmem69
-1,219402381 2,34E-06 Tmem87a
-0,596603436 5,12E-08 Tnni2
-0,62371358 0,000322924 Tnni3
0,656449517 0,001097076 Tns2
-0,653024161 3,20E-06 Tpp1
-1,047339837 0,013770506 Trex1
1,325603164 0,000178927 Trim63
-0,750117629 0,000254022 Tsc22d3
-1,443546061 0,000951947 Tuba3a
-0,699132038 0,000145423 Tuba4a
-0,684589323 0,000479482 U2af2
-1,140448135 0,042490366 Ucp1
-0,626847192 0,003094696 Ufl1
-1,015546519 0,000303474 Ufsp2
-0,681567314 0,009282325 Uqcc2
-0,736420366 0,003199699 Uqcr11
0,614920305 0,005438289 Vamp1
-0,687454212 6,74E-05 Vcan
-0,816717731 4,67E-06 Vps26c
-0,800087004 0,000372249 Vrk1
-0,728185019 1,57E-06 Wdfy1
-0,628186988 2,22E-05 Wdr92
-0,988466586 5,10E-05 Wipi2
-0,799067108 0,001355112 Yap1
-1,144191829 0,004989825 Yod1
-0,696911292 9,84E-06 Zbtb8os

1,198283095 7,59E-06 Zfand
