# Supplementary material for: Aberrant mitochondrial dynamics contributes to diaphragmatic weakness induced by mechanical ventilation
Source: PNAS Nexus. 2023 Nov 7;2(11):pgad336. doi: 10.1093/pnasnexus/pgad336 (PMC10635656; doi:10.1093/pnasnexus/pgad336)
Supplement: pgad336_Supplementary_Data [file pgad336_supplementary_data.zip › PNASNEXUS-PNASNEXUS-2023-00277RR-s03.pdf]

Supplementary table 2

Column 1: KEGG_JAK_STAT_SIGNALING_PATHWAY
Column 2: KEGG_OXIDATIVE_PHOSPHORYLATION
Column 3: KEGG_CALCIUM_SIGNALING_PATHWAY

PIK3CB	LHPP	PLCB1
CNTFR	COX7B	MYLK
STAT5A	ATP6V1C1	PLCB3
GRB2	PPA2	PLCG2
STAT1	PPA1	CAMK2B
AKT1	ATP6V1H	CD38
PTPN6	ATP6V1D	ATP2A2
STAT5B	ATP6V1A	PPP3CB
AKT2	ATP6V1E1	PRKACB
STAM	SDHD	GNAS
PTPN11	ATP6V1B2	CHP1
CTF1	SDHA	ATP2A3
STAT3	ATP6V0D1	NOS1
PIK3R1	NDUFB2	PPID
LIFR	ATP4A	CAMK2G
	NDUFA2	TNNC1
	NDUFV1	PPP3CA
	SDHB	MYLK2
	UQCRFS1	VDAC3
	ATP5PB	ATP2B1
	NDUFS1	PLCD1
	UQCRC2	PLCG1
	NDUFB9	CACNA1S
	NDUFB4	CAMK2D
	CYC1	PHKB
	UQCRC1	PTK2B
	NDUFA10	RYR1
	NDUFS2	GNAQ
	NDUFAB1	VDAC2
	NDUFB6	EGFR
	NDUFB5	PRKACA
	NDUFA5	VDAC1
	NDUFS8	SLC25A5
	UQCRQ	PLCD4
	COX5B	ATP2A1
	NDUFA7	ATP2B4
	SDHC	BST1
	NDUFB7	PHKG1
	NDUFS7	GNA11
	NDUFA9	PPP3R1
	UQCRB	PRKCA
	COX6A2	TNNC2
	NDUFB3	PHKA1
	NDUFS3	SLC25A4
	COX7A1	CAMK2A

NDUFA6 SLC25A31
COX5A
NDUFC2
NDUFB10
NDUFS4
UQCR10
ATP6V1G1
NDUFA3
COX7A2L
NDUFV2
NDUFA8
NDUFA1
MT-CO3
COX11
UQCRH
COX6B1
COX7A2
NDUFB8
NDUFV3
NDUFS6
NDUFA11
COX4I1
COX6C
NDUFA4
COX7C
COX15
NDUFS5
ATP6V1G2
UQCR11
COX17
