# Supplementary figures and images for: Aberrant mitochondrial dynamics contributes to diaphragmatic weakness induced by mechanical ventilation
Source: PNAS Nexus. 2023 Nov 7;2(11):pgad336. doi: 10.1093/pnasnexus/pgad336 (PMC10635656; doi:10.1093/pnasnexus/pgad336)

**A**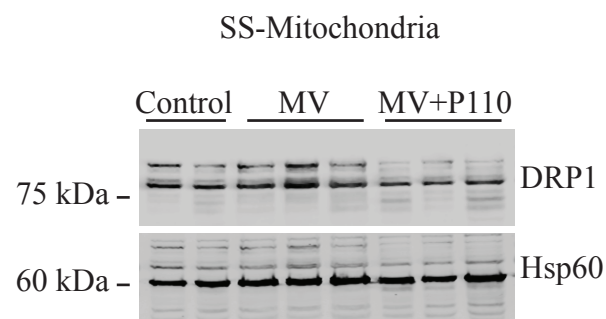**B**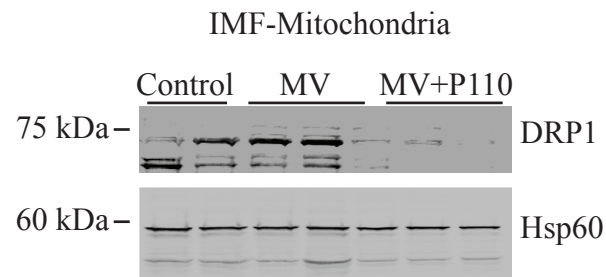**C**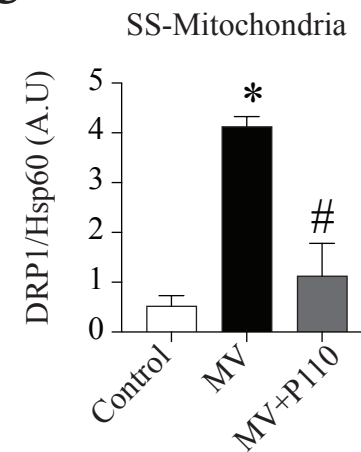**D**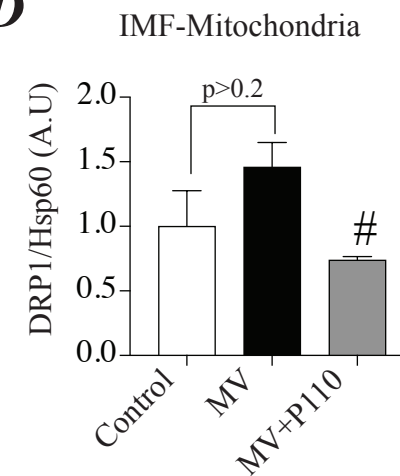**E**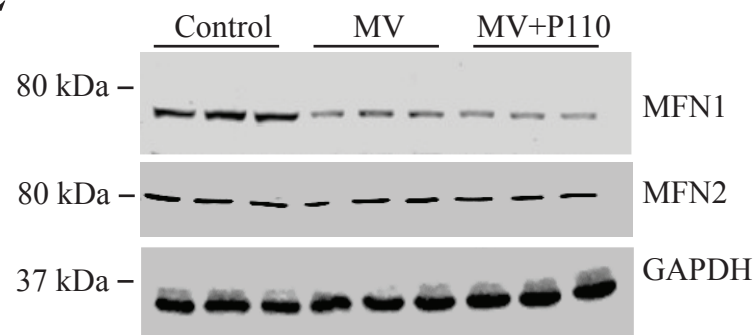**G**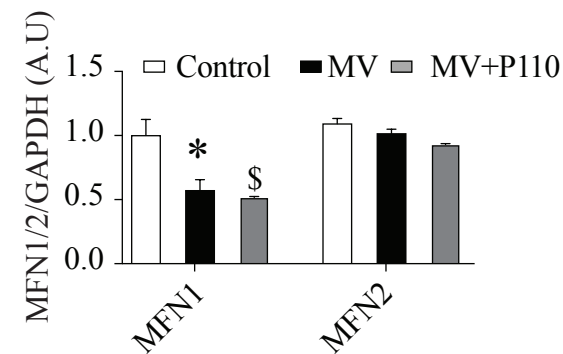**H**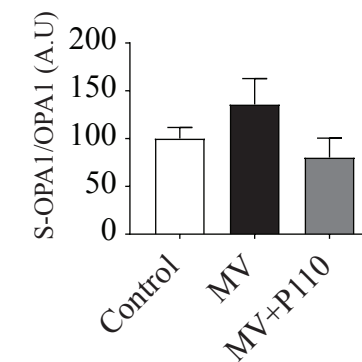**F**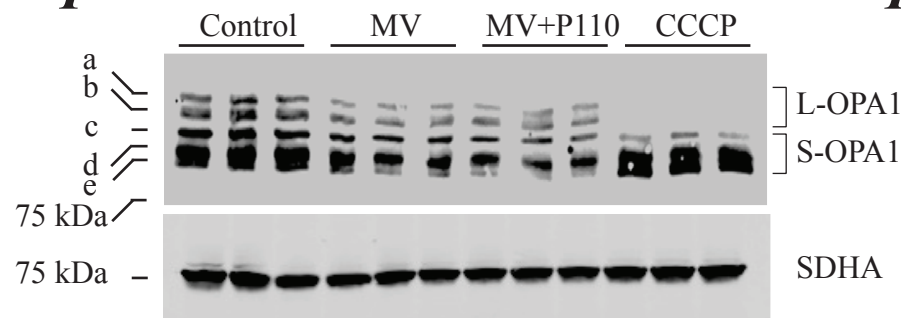**I**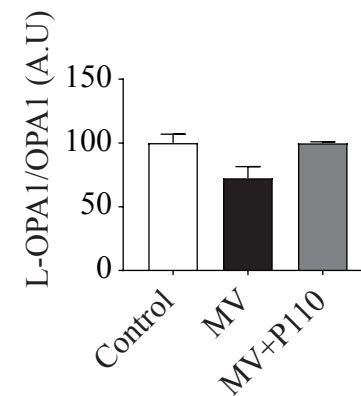**J**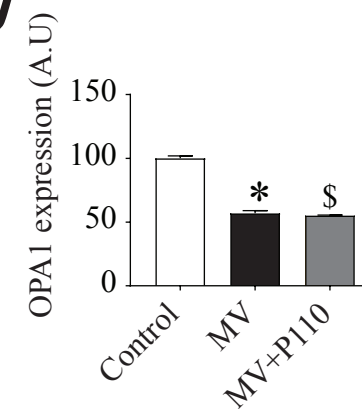

Supplement: pgad336_Supplementary_Data [file pgad336_supplementary_data.zip › PNASNEXUS-PNASNEXUS-2023-00277RR-s01.pdf]
